# 

**Published:** 1895-07-06

**Authors:** 


**Notices of Births, Deaths, and Marriages** are inserted in this column at a charge of 2s. 6d. for an announcement not exceeding four lines.

**Notice to Advertisers and Subscribers.**

All Advertisements, Orders for Copies of THE HOSPITAL and Business Communications should be addressed to **The Manager, NOT TO THE EDITOR,** THE HOSPITAL, 428, Strand, London, W.C.
The Cost of Subscription, post free, is as follows:—Per week, 2½d.; per quarter, 2s. 8d.; per half-year, 5s. 3d.; per annum, 10s. 6d. Foreign:—Per Annum, 15s. 2d.; payable, in all cases, in advance.

### NOTICE TO CORRESPONDENTS.

All MS., Letters, Books for Review, and other matters intended for the Editor should be addressed THE EDITOR, THE LODGE, PORCHESTER SQUARE, LONDON, W.
The Editor cannot undertake to return rejected MS., even when accompanied by stamped directed envelope.

**"Burdett's Hospital Annual," 1895,**
NOW READY.
Sixth year of publication. 950 pp. Scarlet cloth, gilt lettered. Price 5s. A most complete guide to British, Colonial, Indian, and American Hospitals, Dispensaries, Nursing and Convalescent Institutions, and Asylums. A few copies of the "Annual" for 1894 may still be obtained on application to the Publishers, THE SCIENTIFIC PRESS (Limited), 428, Strand, London, W.C.

**NOTICE.—Vol. XVII. of "The Hospital" (October, 1894, to April, 1895), in handsome cloth binding, is on sale at the Office of this Journal. Price 6s. Cases for binding the Numbers contained in this Volume, 1s. 6d. Index to Vol. XVII., 2½d., post free.**

### Scraps and Gleanings.

Purses amounting to £260 were received by H.R.H. the Duchess of Teck at the opening of the Mary Adelaide Ward at the Chelsea Hospital for Women.

By the will of Mrs. Anne Tourz Hall a bequest of £20,000 comes to St. George's Hospital, the testatrix leaving the same as a memorial testimony to her late husband.

The annual church parade of the United Friendly Societies of Okehampton has just been held. The collections, amounting to £15, have been forwarded to the Devon and Exeter Hospital.

Dr. Jean Charcot, son of the celebrated French specialist, has received his diploma of Doctor of Medicine. Dr. Charcot, we learn, intends carrying on his father's special line of investigation.

The German Emperor has promised to give a large sum of money and a site for the proposed monument to Helmholtz to be erected at Berlin. For this object international contributions are being solicited.

The committee of the Corney Grain Memorial Fund announce subscriptions already received of upwards of £500 towards the endowment of the cot in the Hospital for Sick Children, Great Ormond Street.

Last year the Ladies' Committee of the Hull Infirmary obtained £530 through the efforts of their Saturday Fund; this year again every effort is being made to ensure a like success in the same scheme for the good of the local hospitals.

During the afternoon which was devoted to the Hospital Sunday Fund collection in Birmingham, Alderman Cook, who has given so much of his time to the well-being of the fund, was presented with an illuminated address by the Executive Committee.

The Duke and Duchess of York have consented to receive a deputation from the Victoria Hospital for Children at York House, St. James', who will present to their Royal Highnesses a cot endowed in perpetuity, in commemoration of the birth of Prince Edward of York.

A bazaar has lately been held locally to swell the fund inaugurated for the purpose of supplying the poorer classes who have to attend the Royal Infirmary at Hull with splints, bandages, &c., and other necessary appliances they may require, accessories which are beyond the means of many of the patients.

Quite recently a special service was held in the chapel of the Devon and Exeter Hospital on the occasion of the dedication of a marble memorial tablet which has been fixed at the west end of the chapel to the memory of the late Rev. W. Hocken, who was for forty-five years chaplain to the hospital.

Professor Verneuil, who died recently in his country house, near Paris, at the age of 72, ranked among the ablest surgeons in France, and was for more than 20 years Clinical Professor to the Faculty of Paris, having served as senior surgeon to the Hospital of La Pitié Lariboisière and Le Midi.

Mr. Peter Waddell, a gentleman of means, has left the residue of his fortune, after paying small legacies to relatives, to the Royal Infirmary, the Longmore Hospital for Incurables, and the Royal Blind Asylum, Edinburgh, and the Leith Hospital, in equal shares. The estate is expected to realise about £120,000.

A public meeting has been held in the De Grey Rooms at York to consider a scheme for increasing the usefulness of the York Dispensary. The committee are desirous of establishing a maternity charity in connection with the Dispensary. The sympathetic and local co-operation of the public will be cordially appealed to for this deserving object.

Dr. Barnardo applied to the Town Council of Exeter for permission to start next month street collections at Exeter in favour of funds for his homes for waifs and strays. But as the local Hospital Saturday Fund had previously been fixed for the same time, the Town Council had reluctantly to postpone permission for Dr. Barnardo's scheme.

At the last court of the guardians of the Royal Caledonian Asylum, at Holloway, the chairman appealed for further and more liberal support towards this deserving charity which has maintained and educated no less than 3,000 boys and girls, many of whom were the orphans of Scotch soldiers who had lost their lives in the service of their country.

The committee of the Queen Square Alexandra Hospital for Children with Hip Disease are issuing a special appeal towards the proposed new building fund. A sum of £12,000 will be required for this purpose. The hospital is one deserving a hearty response from the charitable public. Besides being the only one of its kind, this institution has carried on a highly efficient work.

At a meeting of the Liverpool City Council a resolution, to which we have already alluded, was formally carried granting to the trustees of the Northern Hospital two sites adjacent to the present hospital consisting respectively of 630 and 1,545 square yards, and also to give £10,000 to the funds of the hospital. The new hospital is to be built by the David Lewis trustees, and will be on a greatly enlarged site, including part of that now occupied by the present building.

### Books Received.

Chatto and Windus.

"The Moonstone" (Sixpenny Edition). Wilkie Collins.

Horace Cox.

"An Australian in China." G. E. Morrison.

H. K. Lewis.
"Mentally Deficient Children." Shuttleworth.
"Hygiene." Louis C. Parkes.

George Newnes, Ltd.
"The Princess of Wales." Mary Spencer Warren.

J. and A. Churchill.
"Myxœdema and the Thyroid Gland." J. D. Gimlette.

Swan Sonnenschein.
"The Cell." Dr. Oscar Hertwig.

Horne and Son.
"Guide to Whitby."

Bailliere, Tyndall, and Cox.

"Prevention of Consumption." Wm. Murrell.

**Periodicals and Pamphlets.**—Strand Magazine, Picture Magazine, Strand Novelettes, Round the Coast—Part 3, Clinical Sketches. The Corpuscle, The Therapist, Girl's Own Paper, Girl's Own Paper Summer Number, Boy's Own Paper, Boy's Own Paper Summer Number, English Illustrated, London Home Monthly, Sunday at Home, Leisure Hour, Tri-State Medical Journal, Cornhill Magazine, Gentleman's Magazine, Humanitarian, Penny Tales for the People, Guy's Hospital Gazette, Literary Digest, Cassell's Family Magazine.

### The Student of Medicine.

### APPOINTMENTS.

J. Biggam, M.A., M.D.Edin., reappointed Medical Officer of Health to the Sedgley District Council. Dr. Bond appointed Medical Officer of Health for the Holborn District. W. Brown, M.D.Glasg., M.B., C.M., reappointed Medical Officer of Health to the Stapleton District Council. G. H. Fosbroke, M.R.C.S. Eng., B.P.H., appointed Medical Officer for the St. Peter the Great District of the Pershore Union. C. Gordon, M.B., C.M. Edin., appointed Medical Officer to the West Malvern District of the Malvern Dispensary. W. C. Hamilton, M.B., C.M.Edin., appointed Senior House Surgeon to the Blackburn and East Lancashire Infirmary. F. Hunton, M.D.Durh., appointed Medical Officer for the Stockton District and the Workhouse of the Stockton Union. L. Napier, M.D.Aberd., M.R.C.P.Lond., F.R.S.Edin., appointed Examiner in Midwifery to the Society of Apothecaries of London. W. Partington, M.B., C.M.Glasg., reappointed Medical Officer of Health to the Tunstall Urban District Council. J. R. Purdy, M.B., C.M., appointed Medical Officer of Health to the Hutt County Council. M. B. Ray, M.B. Edin., appointed Fifth Assistant Medical Officer to the South Yorkshire Asylum, Wadsley, Sheffield. Mr. Smyth appointed Assistant Medical Officer at the Parish of Birmingham Infirmary. W. H. Thompson, M.D.Durh., L.R.C.P., L.R.C.S.Edin., reappointed Medical Officer of Health to the Quarry Bank Urban District Council. J. A. Turner, M.B., C.M.Edin., D.P.H.Camb., reappointed Medical Officer of Health for Lutterworth. T. P. Whittington, L.R.C.P., L.R.C.S.Edin., appointed Medical Officer of Health to the Neath Rural District Council. T. G. Brodie,

THE STUDENT OF MEDICINE—*continued.*

M.D., L.R.C.P.Lond., appointed Lecturer on Physiology at St. Thomas's Hospital. C. Thompson, M.B., C.M.Edin., appointed Assistant to the Professor of Surgery and Clinical Surgery at the University of Louisville.

### VACANCIES.

#### Resident Medical Officers.

Birmingham General Dispensary.—Resident Surgeon; doubly qualified. Salary £150 per annum, with an allowance of £30 per annum, for cab hire, furnished rooms, fire, lights, and attendance. Applications to Alex. Forrest, Secretary, by July 15th.

Flintshire Dispensary.—Resident House Surgeon. Salary £120 a year, with furnished house, rent and taxes free, also coal, light, water, and cleaning, or in lieu thereof a sum of £20 per annum. A knowledge of Welsh desirable. Applications to Thos. Thomas, Secretary, Board Room, Bagillt Street, Holywell, N. Wales, by July 17th.

Newport and Monmouthshire Infirmary, Newport, Mon.—House Surgeon; doubly qualified. Salary £100 per annum, with board and residence. (No stimulants provided.) Applications to the Secretary by July 13th.

National Sanatorium for Consumption and Diseases of the Chest, Bournemouth.—Resident Medical Officer. Salary £80 per annum, with board, lodging, and washing. Applications to the Secretary by July 25th.

Royal Victoria Hospital, Bournemouth.—House Surgeon and Secretary. Salary £100 per annum, with board. Appointment for two years. Applications to the Chairman of the Committee by July 17th.

Sheffield General Infirmary.—House Surgeon and Senior Assistant House Surgeon, doubly qualified. Salary for the former £120 per annum, with a prospective advance of £10 per year for the second and third years; and for the latter £80 per annum, with board, lodging, and washing. Applications to the "Medical Staff of the Sheffield General Infirmary, to the care of the Secretary," by July 13th. The election will take place on July 26th.

Suffolk General Hospital, Bury St. Edmunds.—House Surgeon. Salary £100 per annum, with board, lodging, and washing. Applications to Henry Bonner, Secretary, by July 15th.

#### Non-Resident Medical Officers.

Dental Hospital of London, Leicester Square, W.C.—Two Assistant Dental Surgeons. Applications to J. Francis Pink, Secretary, by July 8th.

London Hospital Medical College, Mile End.—Senior Demonstrator of Anatomy. Salary payable by a percentage of fees. Applications to Munro Scott, Warden, by July 8th.

London Hospital Medical College, Mile End.—Senior and Junior Demonstrators of Physiology. Salary for the senior post £150 a year and a proportion of the fees paid for classes, and for the junior post £50 a year. Applications to Munro Scott, Warden, by July 8th.

Middlesex Hospital, W.—Assistant Physician; must be F. or M.R.C.P. Lond. Applications to F. Clare Melhado, Secretary-Superintendent, by July 8th.

### THE SOCIETY OF APOTHECARIES OF LONDON.

The following changes have taken place in the staff of Examiners of the Society of Apothecaries of London: Mr. Bilton Pollard has been appointed one of the Council Examiners in Surgery, vice Mr. A. Clark, who retires by rotation. Mr. C. B. Lockwood has been appointed one of the Society's Examiners in Surgery, vice Mr. Bilton Pollard. Dr. T. D. Savill has been appointed an Examiner in Medicine, vice D. F. Warner, who retires by rotation; and Dr. Leith Napier has been appointed an Examiner in Midwifery, vice Dr. Lewers, who retires by rotation. Mr. Stonham has been appointed an Examiner in Anatomy, vice Mr. C. B. Lockwood.

Pass List, June, 1895.—The following candidates passed in:—

*Surgery.*—J. Blackwood University College; F. H. Blatchford, St. Mary's Hospital; F. E. Bromley, London Hospital; B. L. Dhingra, Lahore; A. P. Eldred, St. Mary's Hospital; A. H. H. Huckle, Guy's Hospital; W. B. Maurice, St. Mary's Hospital; A. H. Palmer, Birmingham and Guy's; R. G. Worger, Bristol and Guys; A. M. St. J. Wright, Madras.

*Medicine, Forensic Medicine, and Midwifery.*—J. Blackwood, University College; M. A. Cooke, St. Bartholomew's Hospital; R. A. Fegan, St. Bartholomew's Hospital; A. H. Palmer, Birmingham and Guy's; C. J. Palmer, Birmingham and Liverpool.

*Medicine and Midwifery.*—J. W. Clegg, Manchester; R. B. Rees, Middlesex Hospital.

*Medicine.*—E. A. B. Poole, Birmingham.

*Forensic Medicine and Midwifery.*—W. A. Montgomery, St. Thomas's Hospital; C. Blue, Belfast.

*Forensic Medicine.*—H. G. C. Hardwick; Cambridge and St. Thomas's Hospital; J. A. Morgan, Charing Cross Hospital; R. G. Worger, Bristol and Guy's Hospital.

*Midwifery.*—T. H. Wilkins, Charing Cross Hospital.

To Messrs. Blackwood, Cooke, Clegg, Fegan, Hardwick, Huckle, Morgan, Palmer, A. H. Rees, and Worger was granted the diploma of the Society entitling them to practise medicine, surgery, and midwifery.

At the Examination in Arts held this month 18 candidates passed, of whom one was placed in the first class. Six candidates passed in certain subjects only.

